# Selection of Person of the Year from Public Health Perspective: Promotion of Mass Clusters of Copycat Self-immolation

**Published:** 2012

**Authors:** Mehran Zarghami

**Affiliations:** 1 Psychiatry and Behavioral Sciences Research Center and Department of Psychiatry, Mazandaran University of Medical Sciences, Mazandaran, Sari, Iran

## Abstract

Suicide is a complex phenomenon with multiple diverse causes. It is obvious that if the media give rewards to the suicides directly or indirectly, they will have more *copycat effect. *It seems that political means in the mainstream media, particularly in the Western world overshadow public health- especially mental health- issues. Selection of a Tunisian fruit vendor who set himself on fire in a public square as the person of the year by Time Magazine is a good example in this domain. Mass clusters of copycat altruistic self-burning promotion in the Middle East and North Africa obviously demonstrate the importance of the dilemma and its relevance in terms of public health, and the need to develop efficacious interruptive strategies able to prevent people from committing a dramatic fatal act.

Suicide has been defined as a fatal act which is deliberately performed by a person in the expectation of its fatal outcome (1). This is a complex phenomenon with multiple and diverse causes (2-4). Several suicide preventive strategies are based on medical, ecological and sociological conceptual models (5). The sociological model goes back to *Durkheim*’s classic categorization of suicides as anomic, altruistic, egoistic and fatalistic (6). Despite its limitations, Durkheim's practice has influenced proponents of control theory (7). Durkheim has explained suicide *sociologically* within a holistic perspective instead of particular individual explanation. He has emphasized at variation among social environments in the incidence of suicide (8, 9). 

*Altruistic suicide* in his viewpoint occurs in highly integrated societies, where the society's needs are seen as more important than individual’s needs, and the individuals kill themselves on behalf of the society (10). However, there are controversies on underlying relations in Durkheim's work. For example, Berk believes that psychological and individual variables as non-social causes of suicide, overlook social forces (8). 

Documented evidence suggests that one of the underlying causes of suicide may be a maladaptive by-product of generally adaptive social learning principles (4, 11). 

Some people imitate another suicide that they know about either from local knowledge, having a close relationship or from the media. Following Goethe's novel “*Die Leiden des jungen Werthers*“ (*The Sorrows of Young Werther*) in 1774- in which many men replicated the act of the hero (shooting himself) - (12) the effect of this knowledge is called “*Werther effect*” (13). This process is called “*suicide contagion*” (14), and this kind of suicide is known as “*copycat suicide*” (15, 16). It seems that some myths (i.e. wrong judgments concerning suicide that are culturally confirmed and do not have any scientific basis) (17) may be expanded through media. 

Some copycat suicides spread through clusters of people (e.g. schools, communities, nations and regions). These are called “*suicide clusters*” (14, 15). Two common patterns of suicide clusters have been documented (4, 18) Copycat cluster suicides which occur in both the same time and the same space are called “*point clusters*” and those which occur in the same time but not the same space are called “*mass clusters*” (18). Point clusters have been attributed to social learning within local groups (19), and mass clusters have been attributed to prestige and similarity bias (11) and the mass media (20, 21). 

Recently, Rezaeian has discussed the unique pattern of suicide cluster and introduced a novel type of categorization. He states that the dilemma with the previous categorization is that the important role of place or space is overlooked in a mass cluster. In other words, under such situations, the cluster finally encompasses a wider geographical area which is covered by the media (22).

Many researches have cited the mass media (newspapers, magazines, television, radio etc.) as a trigger of mass clusters of suicide (20,21,23). The internet and satellite television have increased the global range of the mass media in recent years (4). Mass clusters are typically related to high-profile celebrity suicides that are broadcasted in the public mass media (4,23,24). In other words, widely publicized celebrity suicides are more likely to precipitate a copycat suicide (23,25,26). 

Examples of celebrities whose information had been publicized and disseminated via mass media and have inspired suicide clusters include Ruan Lingyu, the Japanese musicians Yukiko Okada and Hide and Marilyn Monroe, whose suicides was followed by an increase of 200 more suicides than average for one month (14, 15). 

It seems that a copycat suicide may have anomic, altruistic, egoistic or fatalistic nature, and the nature of the first (original) suicide may not be necessarily the same as the copycat ones. For instance an anomic or egoistic suicide may be imitated altruistically, vice versa.

At least 120 scientific studies were published on the possible association between media descriptions of suicide and actual copycat suicides between 1967 and 2009 (25). This fact has been confirmed after controls for confounding variables (such as unemployment trends, season, holiday and day of the week effects) in large scale investigations (25). 

According to the “*laws of imitation*”, mass clusters of suicide result from two social learning biases: *prestige bias*, where people prefer to copy the behavior of high-status and/or reputable models (4, 27), and *similarity bias*, where people prefer to copy the behavior of models that resemble to them in ethnic characteristics (4,11,28). In other words, some people believe that similar people face similar challenges and same stressors, and so may have suitable solutions to such challenges (4,25,28). These are called “*vertical*” and “*horizontal*” *identification* respectively (25,26,29). 

This is an important fact that vertical and horizontal identification may occur together, and both celebrity and no celebrity publicized suicides are often associated with increases in the copycat suicides (25,30,31). 

It should be emphasized that the immediate rise in suicide rate is not caused by already-vulnerable people committing suicide earlier than they otherwise would have. Because evidence shows that suicide rates do not drop some time after the publicized suicide (4, 24). Also besides the characteristics of the stories and the audience, it has been documented that, the greater the coverage of a suicide by the media (such as the number of networks or newspapers carrying the story of suicide, the priority of newspaper pages and the column inches, seconds of radio and TV news etc.), the greater the Werther’s effect (25). 

On the other hand, people and groups with pre-existing protective factors against suicide will be less predisposed to Werther effects, and people with some risk factors are more vulnerable (25). 

Media representations of suicide are a basic component of social learning processes, and may have positive and negative effects to their audience (23, 25, 32-35). Positive definitions of suicide encourage a copycat effect, and negative definitions minimize the odds of this effect. However, media coverage usually has more positive and/or neutral approach (25). 

Positive approach to suicide includes individual and social rationalizations, giving attention and rewards, focusing on the positive aspects of victim’s life and glorification of deceased person (25). 

Negative approach to suicide includes emphasizing that suicide is wrong, valueless, and even foolish; focusing on victim’s injury and deformity/disfigurement; arguing alternative solutions, and discouragement of suicide by shaming the victims (36). Emphasizing on negative definitions of suicide in suicide stories is 99% less likely than their counterparts to report a copycat effect (37). Well-known dishonored figures that have negative prestige (e.g. War spies) and non-celebrities, both of whom lack reputations, have non-significant or smaller effects on suicide rates (4, 23, 26).

Using scan statistic methods and agent-based simulations to clarify the social learning processes underlying point and mass clusters found that point clusters are generated by social learning between neighboring agents. This effect was sometimes mimicked by a phenomenon called “homophily”, which means that people prefer assorting with similar others. Mass clusters are formed by one-to-many transmissions by the mass media, perhaps due to copying prestigious celebrities (prestige bias) and/or only copying similar models (similarity bias), where social learning was weak (4). 

Media-publicized suicides have resulted to increased suicide rates in many countries, including Austria (38), Japan (39), Germany (40), and Taiwan (41). 

*Deliberate Self-Immolation*

Deliberate *self-immolation* is the most dramatic, violent and often the most difficult to understand method of suicide.

The word self-immolation in Latin-based English means sacrifice oneself, without any reference to burning (42). 

Self-immolation has been practiced for many centuries, especially in India. This phenomenon is admitted by some principles of Mahayana Hinduism and Buddhism for various reasons, including renouncement, Sati, devotion, and political protest. Some warrior cultures, such as in the Rajputs and Charans, also practiced self-immolation (42). 

Western media gave a strong association between "self-immolation" and fire, and introduced this word to a wide English-speaking audience after coverage of Buddhist monks setting fire on themselves in protest of the South Vietnamese regime in 1963 (42). 

In modern times, “*self-immolation*”, “*self-burning*” or “*Bonzo*” has become a type of radical political protest (42). 

Self-immolations are often newsworthy, because of their public, dramatic, and political nature. They attract attention and become aggrandized as martyrdom, because of the perception of great discomfort. They may be perceived as a type of altruistic suicides for the collective effects (42). 

Instances of deliberate self-burnings had been reported for centuries in Vietnam, including multiple cases during the 1920s and 1930s. Also this practice had been recorded in 1948 in Harbin (China) when a monk immolated in protest against the communists of Mao Zedong (43,44). Another case had been seen in 1950 in North Vietnam (44). 

A number copycat self-immolations occurred by Buddhist monks who burned themselves in protest of the persecution of Buddhists by South Vietnam's Roman Catholic government. The most famous of them was a Vietnamese Buddhist monk named Thích Quảng Đức, who burned himself to death at a busy Saigon road intersection in 1963. His heart which had been remained intact (44-46) was considered to be holy and placed in a glass chalice and was interpreted as a symbol of compassion, amplifying the impact of his death on the psyche of the world public (44, 47). He was called a “*martyr*” and Photos of his self-burning were quickly circulated widely and were featured on the front pages of newspapers worldwide. Malcolm Browne (the photographer) won a Pulitzer Prize, and another similar photo won the World Press Photo of the Year in 1963 (48). 

Five more copycat self-immolations occurred after Đức by Buddhist monks until late October 1963 as the protests escalated in Vietnam (46). 

A famous self-immolation event took place in Tianmen Square in China, in which five members of Falun Gong, a prohibited movement, set themselves on fire to protest the unfair treatment of their spiritual band by the Chinese government in 2001 (49, 50). The video of the incident was broadcasted (51) and received prominent international news coverage (49). 

As mentioned above, it has been documented that self-immolations have led to numerous copycat suicides. Almost 100 self-immolations have been presented by the *New York Times* and *The Times* between 1963 and 1971 (44, 52), most of them happened in the United States protesting the Vietnam War and Asia. Đức's act was followed by American citizens in protests against the Vietnam War, and by other monks for other reasons (44, 46). Also this practice occurred in the Soviet bloc by Czech student Jan Palach in 1968. Non-political self-immolations also became more prevalent in these years. In addition, more than 1,584 deliberate self-immolations have been occurred in 2000 and 2001 in India (42, 53). A critical high wave has been reported in this country protesting the Reservation in 1990 (54). 

Also self-immolation was the selected way of Tibetans to protest Chinese rule in early 2011. Sixteen copycat self-immolations have been recorded in this region since 2011 (42). 

The most resent famous case of self-immolation was a Tunisian street vendor named Mohamed Bouazizi )locally known as "Basboosa," (55, 56), who set himself on fire on December 17, 2010 (42) in protest of the mortification that he reported was inflicted on him by municipal officials (15, 56). The Tunisians’ anger aggravated and their revolution precipitated following Bouazizi's suicide, leading then President Zine El Abidine Ben Ali to step down on 14 January 2011, after 23 years in power (56). 

Several reward systems were activated after Bouazizi's suicide. More than 5,000 persons participated in the several funeral processions (57). Public media services such as YouTube and Face book marked images of riots and commemorations (58). Many people in the North Africa and Middle East honored Bouazizi as a hero (59, 60). Mohamed Bouazizi was considered as "*a symbol for eternity*" (56, 61) and a "*hero for Tunisians and the Arab world as a whole*" (59) by Tunisian film directors, and assumed a martyr by the Progressive Democratic Party (PDP) of Tunisia (62). The mayor of Paris named a square in Paris as a tribute to honor Bouazizi (63), and the main square in Tunis was renamed as “Bouazizi Square” posthumously (64). Bouazizi was awarded Sakharov Prize as "*one of five representatives of the Arab people*" in 2011 (65). The new Tunisian government respected him with a postal stamp (56). Some Arab commentators called those men and Bouazizi as "*heroic martyrs*" (66) and their comment were illustrated by the media broadly. At this time, the* Time* magazine named Bouazizi as person of the year 2011 (67, 68). 

Bouazizi's deliberate self-immolation that was rewarded in numerous ways and was covered extensively by the media, acted a catalyst for Tunisian Revolution and triggered protests in several other North Africa and Arab countries. Meanwhile, several men did the copycat suicide and emulated Bouazizi's self-burning (42). 

Since the Holly Qur’an says, “Do not expose yourselves to ruin through you own hands” (69), and violence against oneself is prohibited by the “Hadith” (70), suicide is forbidden and considered as a sin by most interpretations of these facts and Bouazizi's self-immolation resulted controversy among some Muslims. While al-Azhar, the most prestigious religious institution in North Africa and Sunni Arab Middle East countries issued a directive stating "suicide violates Islam even when it is carried out as a social or political protest", some protesters speak sympathetically of self-immolators (66).

Despite the very strong condemnation of suicide by Islam, it seems that we are now seeing a trend: Triggering the protests by self-immolations in some Islamic countries (71). 

Bouazizi's action that was resonated across the region and sparked a wave of copycat self-immolations, is currently ongoing in the North Africa and Middle East, with many self-immolations in Algeria, Egypt, Saudi Arabia, Mauritania, Syria, and Ethiopia (42, 56, 72-74), 13 of them occurred within 13 days shortly after Bouazizi’s suicide (75). Also the wave of copycat self-immolations reached Europe in a few months (56). 

*Media impact*

 The events have shared the extensive use of social media (76, 77), considering a terrifying, serious way that immediately attracts attention, disgust, but also sympathy (77). It is obvious that if the media give rewards to the suicides directly or indirectly, they will have more *Werther copycat effect *(15). In addition to rewards, also criticizing copycat suicides and hearing about them in the media seems to give permission to those who are vulnerable (15, 78). 

According to Werther effect, the majority of the suicides will occur in the same or a similar way as the one publicized. People who are more similar to the person in the publicized suicide are more likely to commit suicide (12, 15, 79, 80). Especially if the publicized suicide is of someone in a similar situation as them, many people believe that action is appropriate for them as well (15). 

Sensationalizing and romanticizing the reports especially about reputations, magnifying the deceased, suggesting that there is an epidemic, simplifying the reasons, and publishing the means of suicides in public media all lead to increases in the suicide rate (15). 

Getting a lot of attention, sympathy, and concern to the deceased person that he/she had never got in life, will propagandize suicide as a glamorous ending. Scientific studies have shown that real stories are more likely to uncover a copycat effect than fictional stories; and televised stories are less likely to report a Werther effect than newspapers’ statements. Another possible factor is its disappointing effect. Vulnerable people may feel that "If they couldn't cut it, neither can I" (81). 

Furthermore, suicidal media content may have an “*indirect Werther effect*”; i.e. impact on others which, in turn, can influence one person´s own future (15, 82). 

Social proof model is an alternative approach to clarify Wherther effect (83). In this model, people imitate those who seem similar demographically, despite or even because of societal disapproval (15). 

*Preventive Mission*

From a public health perspective, suicide prevention within psychosocial approach is one of the most important issues. Several descriptions and proposals promise programs for the prevention of suicide behaviors (15), i.e. establishment of regulations, guidelines and safeguards regarding the news casting of suicides in the media to restrict the glorification and dissemination of suicides (4, 20, 21). 

These guidelines should focus on both the audience characteristics and the story characteristics in mapping media effects (25). The popular media should limit graphic and unnecessary depictions and minimize the unnecessary coverage of suicides to reduce suicide rates.

Health care professionals should treat properly with the facts and information about suicides, especially regarding authority figures and famous people. 

Journalists have an important role in deglamorizing information, illustrations and interpretations about suicide in the journals, newspapers, TV and radio. 

Some organizations have produced guidelines for the reporting of suicide since the mid 1990’s. World Health Organization (WHO) has issued booklets in Preventing Suicide. A resource series are addressed to specific professional and social groups including general physicians, media professionals, teachers, primary health care workers, prison officers, survivors, counselors, at work, and first line responders (84). Also Int’l Association for Suicide Prevention has issued a Resource for Media Professionals in this domain (25). 

*The WHO Media Guidelines include:*

- 1. Take the opportunity to educate the public about suicide
- 2. Avoid language that sensationalizes or normalizes suicide, or presents it as a solution to problems
- 3. Avoid prominent placement & undue repetition of stories about suicide
- 4. Avoid explicit description of the method used in a completed or attempted suicide
- 5. Avoid providing detailed information about the site of a completed or attempted suicide
- 6. Word headlines carefully
- 7. Exercise caution in using photographs or film footage
- 8. Take particular care in reporting celebrity suicides
- 9. Show due consideration for people bereaved by suicide
- 10. Provide information on where to seek help
- 11. Recognize that media professionals themselves may be affected by stories about suicide (85).

Mesoudi advices media to follow WHO,  and others confirmed directions for coverage of any suicide: using extreme limitations in covering these deaths - limit the number of stories, do not romanticize the death, and keep the word "suicide" out of the headline (86). Some countries have national journalism codes to discourage suicide reports (15). For example a strict rule in Norway states that "suicide and attempted suicide should in general never be given any mention", or a more moderate one which mentions that "in cases of suicide, publishing or broadcasting information in an exaggerated way that goes beyond normal dimensions of reporting with the purpose of influencing readers or spectators should not occur". In Turkey films, visual images and pictures depicting suicide cases should not be made public (87). Some other countries do not have national codes but do have in-house similar guidelines. For example in the United States of America there are no industry wide standards or there exists voluntary industry codes in some newspapers or journals which are very generic or there are no industry wide standards in others. In Ireland regulations were introduced on the reporting of suicides recently, which attempt to eliminate any positive connotations the act might have (for example using the term "*completed suicide*" instead of "successful suicide" when describing a suicide attempt which resulted in a death) (15). In Canada, reporting of the details of suicide is discouraged (88). To my knowledge, there are no national journalism codes regarding suicide reports in Iran. 

Australia is one of the few countries where is interested to teach journalism students about reporting suicide related issues. Media Wise is a media ethics charity that provides training for journalists on this subject (15). 

In Ireland a media monitoring program named “Headline”, has provided a National Strategy for action on Suicide Prevention. This program also serves the public to become involved in monitoring the Irish media on issues related to suicide (15).

*Does the Media Follow the Guidelines?*

Although regulations, guidelines and safeguards have been established and disseminated by many organizations including WHO, Centers for Disease Control (CDC), *American Foundation for Suicide Prevention* (AFSP), and American Association of Suicidology (AAS) (25), inappropriate coverage in the suicide news has been criticized. Cross sectional research frequently suggests a gap between principles and components of the guidelines and actual press coverage (25). A survey of in-house guides in the USA showed that only 3 of 16 daily newspapers mentioned the word *suicide* and none of them gave regulations about publishing the method of suicide. As stated the online director of the American Society of News Editors (ASNE), "most ethical decisions are left to individual editors at individual papers ". He indicates that "the industry would fight any attempt to create more specific rules or standards, and editors would no doubt ignore them" (89). 

It seems that political means in the mainstream media, particularly in the Western world overshadow public health- especially mental health- issues. Selection of a person who has committed suicide as the person of the year by Time Magazine is a good example in this domain.

Mass clusters of copycat altruistic self-burning promotion in the Middle East and North Africa obviously demonstrate the importance of the dilemma and its relevance in terms of public health, and the need for developing efficacious interruptive strategies able to prevent people from committing a dramatic fatal act.

## Notes

**Declaration of Interest:** None.
